# Rare Presentation of Rhino-Orbital-Cerebral Zygomycosis: Bilateral Facial Nerve Palsy

**DOI:** 10.1155/2011/216404

**Published:** 2011-04-06

**Authors:** Alireza Mohebbi, Hesam Jahandideh, Ali Amini Harandi

**Affiliations:** ^1^Otolaryngology, Head and Neck Surgery Department and Research Center, Hazrat-e Rasool Akram Hospital, Tehran University of Medical Sciences, Tehran, Iran; ^2^Department of Neurology, Loghman Hospital, Shahid Beheshti University of Medical Sciences, Tehran, Iran

## Abstract

Rhino-orbital-cerebral zygomycosis afflicts primarily diabetics and immunocompromised individual, but can also occur in normal hosts rarely. We here presented an interesting case of facial nerve palsy and multiple cold abscesses of neck due to rhino-orbital-cerebral zygomycosis in an otherwise healthy man. Although some reports of facial nerve paralysis in conjunction with rhino-orbital-cerebral zygomycosis exist, no case of bilateral complete facial paralysis has been reported in the literature to date.

## 1. Introduction

The most common clinical presentation of zygomycosis is rhino-orbital-cerebral infection. The former, and more common, term “mucormycosis” is familiar to most clinicians. However, most mycologists prefer the term “zygomycosis” since other members of this class of fungi can also cause infection in the order Mucorales. Almost all patients with invasive zygomycosis have some underlying pathology that makes them susceptible to be infected. The most common underlying diseases are diabetes mellitus, metabolic acidosis, treatment with glucocorticoids or deferoxamine, hematologic malignancies, solid organ transplantation, iron overload, acquired immunodeficiency syndrome, injection drug use, trauma, burns, and malnutrition [1]. 

## 2. Case Report

**Figure 1:**
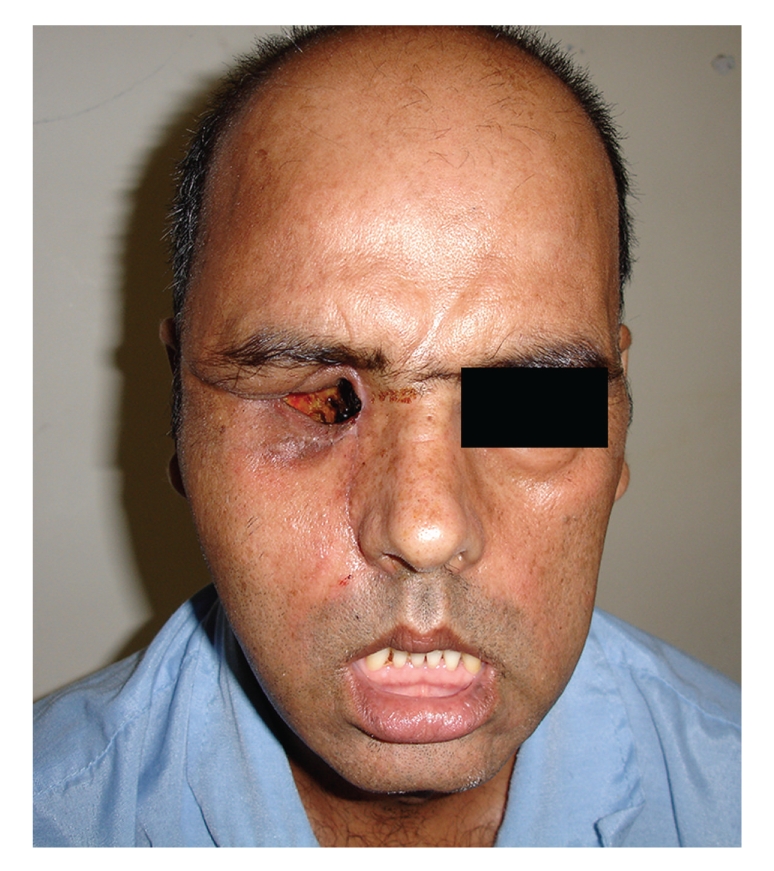
A 43 year-old man affected by zygomycosis and bilateral facial nerve paralysis.

In October 2008 a 43-year-old previously healthy man presented to our hospital with complain of headache, unilateral facial numbness in the distribution area of V1 and V2 branches of trigeminal nerve, facial nerve paralysis, proptosis, blindness due to optic nerve involvement, nasal obstruction, nasal discharge and aural fullness, and hearing impairment in the right side. All these symptoms were developed during a 15-day period. The patient did not have any positive history regarding chronic rhinosinusitis, recent dental problems, or local surgeries. About 48 hours after admission, he developed left-sided complete facial nerve paralysis in all branches (Figure 1). 

**Figure 2:**
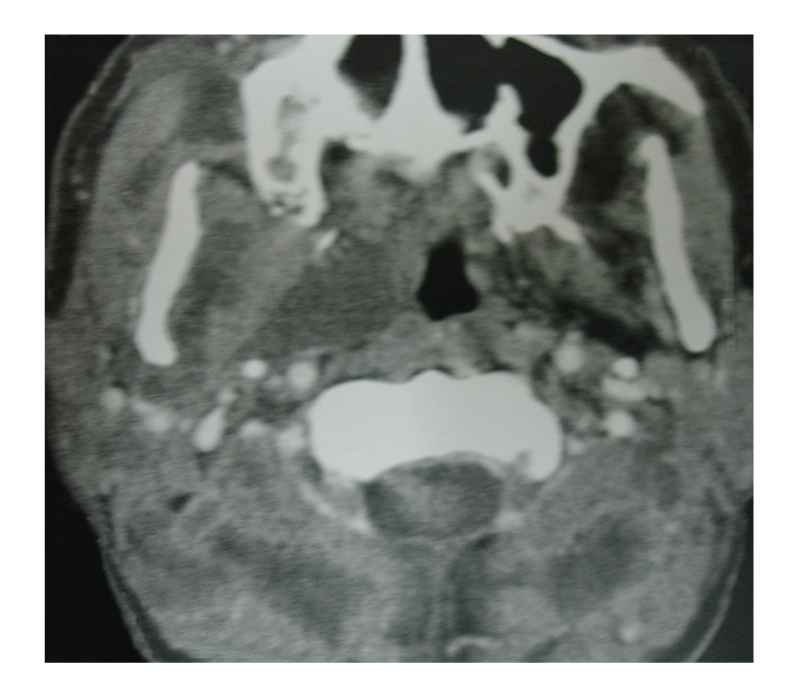
Neck CT scan of the case shows multiple cystic lesions in the neck and right parapharyngeal space.

 Computed tomography scans of orbits, paranasal sinuses, temporal bone, and neck were performed. It demonstrated marked right proptosis, thickening of the extraocular muscles, opacification of right maxillary, ethmoid and sphenoid sinuses, bilateral opacification of mastoid air cells and middle ear spaces, as well as multiple cystic lesions in the neck (Figure 2). According to brain magnetic resonance imaging (MRI) brain stem and cavernous sinus remained intact. Consequently, extraocular muscles involvement was considered as cause of frozen eyes. Except V and VII other cranial nerves were normal. Biopsy of the involved tissues of his right nasal cavity and orbit confirmed zygomycosis. Consequently, patient underwent radical debridement of all involved sinuses, orbital exenteration, incision and drainage of parapharyngeal abscess, and myringotomy and aspiration of middle ear effusion. Histopathological evaluation showed coagulative necrosis, blood vessels thrombosis, and infiltration of broad, nonseptate hyphae with rightangle branching compatible with zygomycosis. Abscess fluid sample was containing fungal elements but middle ear fluid just showed inflammatory changes. Comprehensive immunologic evaluation including measurement of serum immunoglobulins, serum protein electrophoresis, human immunodeficiency virus (HIV) antibodies, antihuman T-lymphotropic virus type 1 and 2 antibodies, lymphocyte transformation time, bone marrow aspiration and trephine biopsy, and flow cytometry for determination of lymphocyte subpopulations was performed. There was a cell mediated immune deficiency with unknown cause including decreased natural killer cells, decreased T helper cells (CD4+) and increased T suppressor cells (CD8+). The patient received amphotericin B up to 3 grams. Results of repeated HIV test after 6 and 12 months were also negative. After one-year follow-up, he has been survived with no evidence of systemic or immunologic diseases or recurrence. Informed consent was obtained from the patient for printing his anonymous photos. 

## 3. Discussion

Rhino-orbital-cerebral zygomycosis is most commonly caused by Rhizopus oryzae [1]. This infection afflicts primarily diabetics and other immunocompromised patients. In rare occasions it can infect normal hosts [2]. Our case had no obvious underlying disorder except a cell-mediated immune deficiency with unknown cause. There are few cases of zygomycosis in the literature secondary to various type of cell-mediated immunodeficiency [3]. Cyst formation in zygomycosis is very rare and has been reported as orbital or brain abscess in the literature [4]. The role of T cells in mediating protection against abscess formation by some bacterial species has been described [5]. However, the protective role of T cells in abscess formation by fungi in the cell-mediated immune deficiency is not clear now. Hearing impairment has been reported in 3.5% of cases in one study [2]. More reports cited facial nerve paralysis in conjunction with rhino-orbital-cerebral zygomycosis [6] with reported frequency of 11% [2]. According to our knowledge, there is no case of bilateral complete facial paralysis in the literature. No obvious pathophysiology for facial nerve paralysis has been proposed yet. Some researchers believe that infection can reach from the pterygopalatine fossa to inferior orbital fissure, orbital apex, and infratemporal fossa [6]. Whether or not the facial nerve is involved at stylomastoid foramen via mentioned pathway is not completely obvious. There is a need to conduct more studies regarding common spread pathways of rhino-orbital-cerebral zygomycosis with presumptive implication in more directed debridement procedures in early stages of the disease.

## References

[1] Cox GM, Basow DS (2009). Zygomycosis (mucormycosis). *UpToDate*.

[2] Ferguson BJ (2000). Mucormycosis of the nose and paranasal sinuses. *Otolaryngologic Clinics of North America*.

[3] Bongiovanni M, Ranieri R, Ferrari D, Codecà C, Tartaro T, Uziel L (2007). Prolonged survival of an HIV-infected subject with severe lymphoproliferative disease and rhinocerebral mucormycosis [14]. *Journal of Antimicrobial Chemotherapy*.

[4] Mnif N, Hmaied E, Oueslati S (2005). Imaging of rhinocerebral mucormycosisL’imagerie dans la mucormycose rhinocérébrale. *Journal de Radiologie*.

[5] Tzianabos AO, Kasper DL (2002). Role of T cells in abscess formation. *Current Opinion in Microbiology*.

[6] Hosseini SMS, Borghei P (2005). Rhinocerebral mucormycosis: pathways of spread. *European Archives of Oto-Rhino-Laryngology*.

